# Radiotherapy for Meningiomas: Molecular, Imaging, and Therapeutic Advances

**DOI:** 10.1007/s11912-026-01766-7

**Published:** 2026-04-21

**Authors:** Bradley Eckelmann, Brett Morris

**Affiliations:** Department of Human Oncology, University of Wisconsin Hospitals and Clinics, Madison, Wisconsin United States of America

**Keywords:** Radiation, radiotherapy, meningioma

## Abstract

Purpose of review: Treatment of meningioma is rapidly evolving, as molecular classification, functional imaging, and radiotherapeutic techniques have undergone recent advances. The purpose of this review is to integrate these developments with clinical trial data to guide modern radiotherapy approaches. We specifically sought to address how new biological and imaging insights inform radiation planning, dose, and outcomes.

Recent findings: Molecular classification provides prognostic insights beyond the classic WHO grading system. DOTATATE-PET has proven valuable for accurate delineation of tumor extent and biologically active regions, directly impacting radiotherapy planning. Recent clinical studies highlight the potential for improved local control with dose escalation and PET-guided planning. Ongoing clinical trials in the intact and salvage settings will provide further insight into the integration of imaging, biology, and radiotherapy.

Summary: Radiotherapy for meningiomas is transitioning into a precision discipline, leveraging molecular and imaging biomarkers for individualized care. Integration of DOTATATE-PET, advanced radiation delivery techniques, and biologically guided dose escalation are redefining standards. Future research will test whether personalized, biomarker-driven radiotherapy can significantly improve outcomes in aggressive and recurrent meningiomas.

## Introduction

Meningiomas are the most common primary intracranial neoplasms, accounting for approximately 40% of all central nervous system (CNS) tumors [1]. The incidence of meningiomas has risen in the United States, in accordance with an aging population and improved access to quality neuroaxis imaging [1]. While the majority of meningiomas are histologically classified as benign (WHO grade 1), atypical (grade 2) and anaplastic (grade 3) tumors can display aggressive clinical behavior, with treatment resistance and high rates of local recurrence [2]. Tumor recurrence or growth in an eloquent location often leads to tumor-specific death. Surgical resection is the mainstay of care, but can be limited in the skull base and along critical optic structures. Current guidelines recommend postoperative radiation therapy (RT) for grade 2 tumors after subtotal resection and for grade 3 tumors after any resection [3]. Select grade 1 tumors can be treated with RT as the primary modality [3]. A variety of RT techniques, included conventionally fractioned RT, fractionated stereotactic RT (FSRT), and stereotactic radiosurgery (SRS) are recommended depending on size and proximity to organs-at-risk (OARs) [3]. In addition, these RT techniques are frequently used in the salvage therapy setting after primary surgery, radiotherapy, or combined therapy.

Over the past decade, genomic profiling, epigenetic studies, and advanced imaging modalities have uncovered a molecularly heterogeneous landscape. These insights have shifted the paradigm away from purely histological grading towards integrated molecular risk classification and image-guided therapy. Consensus efforts, such as the International Consortium on Meningiomas, have begun to propose frameworks for incorporating molecular and imaging data into WHO criteria, though full integration remains ongoing [1].

At the same time, clinical practice has been reshaped by improvements in functional imaging, radiotherapy delivery, particle therapy, and by the exploration of novel radiopharmaceutical agents in clinical trials. Collectively, these developments underscore a shift towards precision medicine for meningiomas. This work aims to synthesize these advances to establish a functional framework for radiotherapy decision-making.

## Molecular Risk Classification

The 2021 WHO classification of CNS tumors recognized molecular markers in meningiomas, with TERT promoter (TERTp) mutations and/or CDKN2A/B deletions signifying grade 3 disease [2]. Recently, this classification was called into question as two large new multi-institutional cohort studies were published [4, 5]. One study found that unadjusted outcomes of TERTp mutated-meningiomas were similar to grade 3 outcomes, but after adjusting for co-occurring alterations, TERTp mutation was not an independent predictor of overall survival, suggesting that poor outcomes in this group are driven by other molecular features that frequently co-occur [5]. This work further suggests that due to the heterogeneity of TERTp-mutated tumors, histologically grade 1 tumors with TERTp mutations would be overtreated if classified according to WHO 2021 criteria. The authors then posit that the utility of TERTp testing may be primarily in grade 2 disease, as TERTp mutants with grade 2 histology behave more aggressively than the general grade 2 population, and can thus be selected for treatment escalation.

The second study found that although TERTp mutation was rare (~ 3%), TERT expression was common (32%) and was a prognostic marker for shorter progression-free survival compared to TERT-negative tumors [4]. They found that TERT expression confers an increase in risk of progression that is roughly equivalent to one grade level, which suggests the use of TERT expression testing may alter treatment decision-making in a higher proportion of patients than TERTp mutation testing.

Other large-scale sequencing efforts have identified molecular profiles that stratify risk more discretely. These have centered around identification of distinct methylation classes, which have repeatedly demonstrated superior accuracy in predicting tumor recurrence and progression compared to the traditional WHO histological grading [6]. Other studies have integrated methylation patterns with DNA copy-number alterations, somatic mutations, and mRNA expression, with similar identification of molecular classes with distinct prognoses that out-predict WHO grading with regard to clinical outcomes [7–9]. The response of these classes to therapy was examined in clinical cases, which found that the magnitude of benefit of adjuvant RT varied across molecular subtypes. The immunogenic class STR observation cohort had a median PFS of 6.5 years, whereas the STR + RT cohort had a 20-year PFS of greater than 75%. In contrast, there was no difference in PFS between observation and RT cohorts in the hypermetabolic, proliferative, or NF2-wt groups [8]. Another study focused on gene expression and identified a 34-gene expression risk biomarker that improved discrimination of outcomes and identified meningiomas that benefited from RT [9]. When combined with surgical criteria into unfavorable or favorable groups, the biomarker could predict RT responses, as unfavorable tumors benefited from RT (HR 0.33, 95% CI 0.14–0.76), whereas favorable tumors did not. These data further suggested that roughly 30% of patients could have had their postoperative management refined by biomarker use. A more recent study found that chromosome 1p loss and 1q gain were independently prognostic of poor outcomes [10]. Grade 1 tumors with 1p loss had similar outcomes to grade 2 tumors overall (median PFS 5.83 vs. 4.48 years); grade 1 tumors with loss of 1p and gain of 1q had similar outcomes to grade 3 tumors overall (median PFS 2.23 years vs. 2.27 years); and grade 2 tumors with 1p loss or 1q gain had similar outcomes to grade 3 tumors overall. This biomarker would reclassify roughly 10% of patients into different risk groups.

**Fig. 1:**
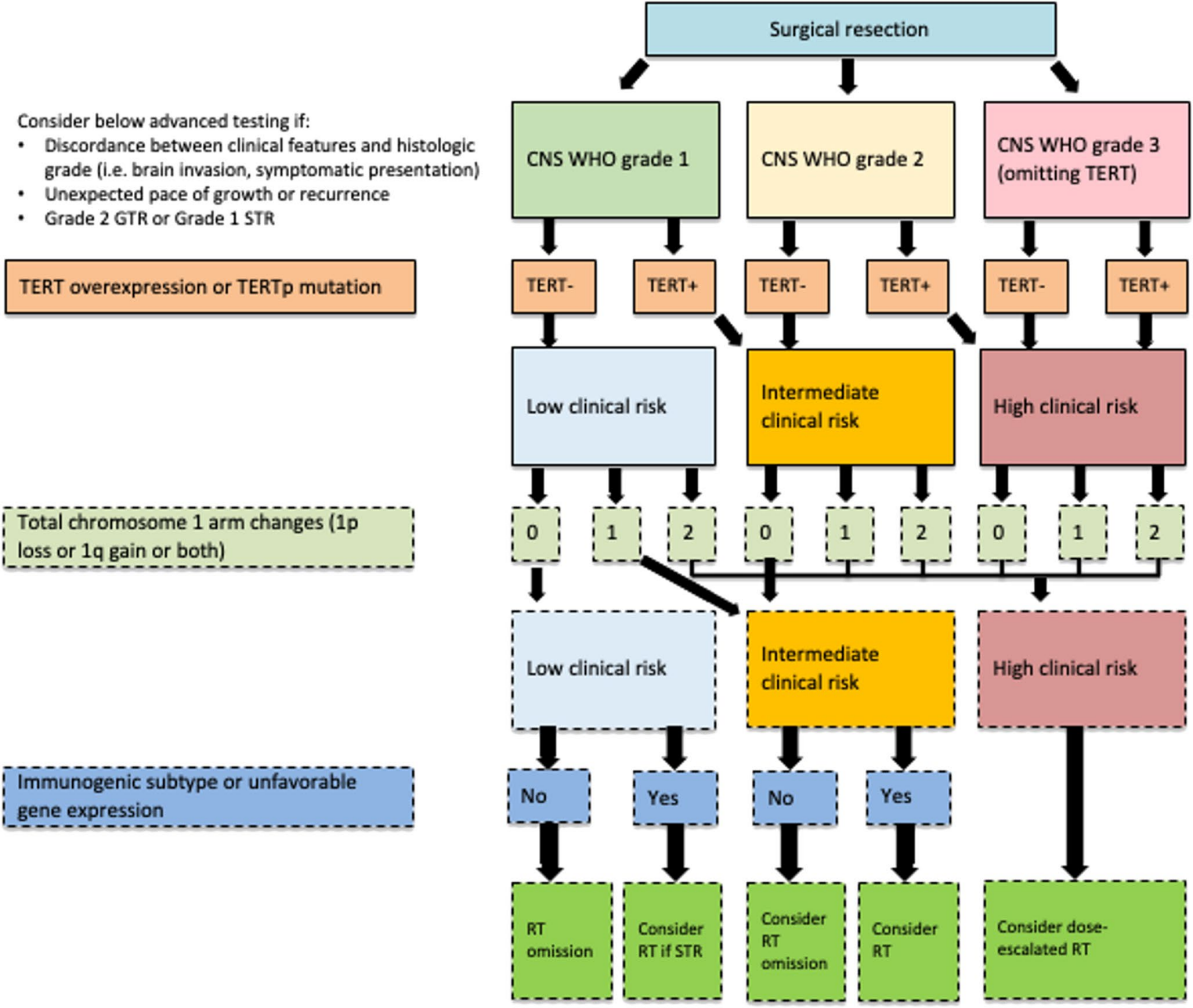
Possible incorporation of molecular risk stratification into radiotherapy decision-making. Testing outlined in solid boxes is recommended by WHO and cIMPACT-NOW guidelines. Testing outlined in dashed boxes is not yet incorporated into guidelines

This work has led to growing advocacy for incorporation of molecular classifiers into clinical workflows. Additional molecular testing has been proposed by working groups for specific clinical scenarios and for future guideline consideration [11]. Additionally, NRG-BN003, which is examining RT after GTR for grade 2 meningiomas, is attempting to validate the prognostic value of a gene expression biomarker [12]. However, EANO guidelines for molecular testing of meningiomas found that no molecular testing targets met their criteria for “ready for clinical use” [11]. Future work is needed to establish the feasibility and utility of these classifiers, whose potential for radiotherapy patient selection is outlined in Fig. 1.

## Radiologic Advances

Radiotherapy for meningiomas has traditionally relied on MRI and CT [13]. CT imaging can detect bone invasion and detail skull base involvement. MRI T1-weighted contrast sequences identify dural-based disease, while T2 and FLAIR sequences provide complementary information about edema and adjacent parenchymal involvement. However, these modalities are limited, particularly in the postoperative or skull base settings where gross disease can easily be mistaken for scar or bone.

Advances in MRI have the potential to improve operative and RT planning [13]. Perfusion imaging can provide maps that correlate with grade and vascularization, and diffusion metrics can serve as surrogates of cellularity. These markers can help define an intracranial lesion as a meningioma, as well as predict histopathologic grade and molecular pathology. Radiomics approaches using multiparametric MRI have shown early promise in predicting recurrence and radiosensitivity.

A major recent advance has been the clinical adoption of PET imaging with radiolabeled somatostatin analogues (68Ga-DOTATATE, among others). Nearly all meningiomas overexpress somatostatin receptor subtype 2 (SSTR2), enabling exquisite tumor-to-background contrast. Several prospective and retrospective studies have demonstrated that DOTATATE-PET is more sensitive than MRI for detecting multifocal disease, osseous invasion, and postoperative residuals [13–15].The benefit of increased sensitivity and specificity may be high in eloquent locations and in demarcation of the dural tail, whether for surgical or radiotherapeutic planning.

The benefits in target delineation and radiotherapeutic planning of DOTATATE-PET are evident. Multiple retrospective analyses have found that PET imaging alters treatment volumes [16–20]. A retrospective analysis of 48 patients found that PET-based volumes differed significantly from MRI/CT-based volumes, with a substantial change in GTV in roughly 70% of cases, primarily reductions in treatment volume [16]. Perlow et al. conducted a retrospective, blinded contouring study that compared PET- with MRI-based contouring, and found that PET enabled more precise radiation treatment volume delineation [18]. This study also found that PET identified new, non-adjacent disease in roughly 30% of cases.

Increased precision may translate into a clinical benefit. Kessel et al. evaluated the long-term benefit of integrating PET imaging into radiotherapy planning for low-grade meningiomas [21]. They found that this workflow significantly improved OS and LC in patients with low-grade tumors. PET-based delineation significantly improved LC on multivariate analysis. Hadi et al. conducted a retrospective cohort study of cavernous sinus grade 1 meningioma examining three groups: surgery alone, surgery and FSRT, and FSRT alone [22]. Patients who underwent RT had PET planning performed. Disease control and functional outcomes strongly favored the FSRT/PET planning cohorts, with 5-year PFS rates of the surgical/FSRT/combined cohorts of 55.7%/100%/100%, and visual toxicity rates of 29%/0%/17%. However, the surgical cohorts had larger tumors and more optic nerve compression at baseline.

Postoperative PET imaging may also allow for more accurate identification of GTR and patient selection for surveillance. A recent study by Ivanidze et al. enrolled patients who underwent GTR identified by PET/MRI managed with surveillance [23]. These patients were compared with a retrospective cohort of patients with MRI-determined GTR, and 5-year PFS was 90% (PET/MR) versus 67% (MR-only).

This imaging modality has now entered consensus recommendations: European Association of Neuro-Oncology (EANO) guidelines and multiple expert groups support SSTR-PET as an adjunct to MRI in cases where delineation is uncertain, especially at the skull base, around bone, or in the re-irradiation setting [11, 24]. Consensus radiation treatment planning guidelines for resected meningiomas utilizing PET have also been published, which recommend smaller CTV expansions and dose escalation of PET-avid disease in certain scenarios [25].

Despite its advantages, clinical implementation of SSTR-PET is limited by accessibility. The biologic information conveyed by SUV values pre- and post-RT has not been explored thoroughly, and may be heterogeneous in high-grade tumors. One small study, which investigated post-RT SSTR-PET SUV and SUV ratio, found a significantly lower risk of progression in tumors which experienced a decrease in SUV ratio or SUV [23]. The utility of PET in the preoperative setting is also unknown and merits exploration. Ongoing clinical trials, including DOTATATE-guided RT studies, aim to standardize thresholds and validate outcomes.

In summary, DOTATATE-PET provides critical complementary information to MRI and CT, which enables radiotherapy plans that are more precise, which may translate to improved disease outcomes.

## Radiotherapy Delivery

Radiotherapy has long been a mainstay in meningioma care, especially for inoperable, subtotally resected, or high-grade lesions. The 2021 EANO guidelines and the NCCN guidelines recommend the use of RT for select grade 1 meningiomas as primary therapy, postoperative RT for all grade 3 tumors, and postoperative RT for grade 2 tumors after STR [3, 11]. After GTR for a grade 2 tumor, both guidelines recommend consideration of radiotherapy.

### Grade 1

In grade 1 disease, the results of observation in the low-risk arm of RTOG 0539 show 3-year PFS after surgery alone is 91.8% [26]. After GTR, 10-year PFS was 87.6%, whereas after STR, 10-year PFS was 72.7%. These results, among others, support select use of observation in this population after successful surgery. In patients who are not candidates for surgery, or the anatomic tumor location makes surgery challenging or high-risk, radiosurgery has emerged as an effective modality for small tumors [27, 28].

The treatment of grade 1 disease with RT is most common when surgery is precluded by anatomic location or patient characteristics. As an example, surgery for perioptic disease is limited, as surgical outcomes have poor local control and high rates of vision loss. Definitive treatment of perioptic (and other eloquent anatomic locations) meningiomas has been explored with various RT fractionation schemes [28]. Hypofractionation, however, is limited by optic apparatus dose constraints. Conventional fractionation has historically had very successful results, with a systematic review and meta-analysis demonstrating pooled LC of 99.8%, with preservation or improvement of vision in ~ 90% of patients [29]. Tumor size was correlated with vision decrement. Estimated rates of radiation-induced xerophthalmia and retinopathy were 10.1% and 7.2%, respectively. FSRT has also been reported to have excellent disease control outcomes, with 5- and 10-year rates of 90.6 and 88.4%, respectively, and minimal toxicity reported in a single-institution retrospective review [30]. Another recent review examined the use of SRS for perioptic disease, finding 5- and 10-year PFS rates of 96 and 89%, respectively [31]. Post-SRS visual decline was found to be 9% at 5 years. Importantly, they discovered that a prescription dose of > = 12 Gy was associated with tumor control. They also found that a Dmax of > = 10 Gy to any aspect of the optic apparatus was associated with visual decline.

Dose limits to the optic apparatus for hypofractionated or single-fraction approaches were recently examined in a recent meta-analysis [32]. This found that Dmax values of 12 Gy in 1 fraction, 20 Gy in 3 fractions, and 25 Gy in 5 fractions were associated with low (< 1%) risks of radiation-induced optic neuropathy.

Grade 1 meningiomas have excellent control rates with SRS, regardless of location. A recent systematic review and meta-analysis reported 10-year LC between 71 and 100%, with marginal doses of 12–15 Gy and low toxicity rates [33]. Overall, recent data continues to support the use of radiation therapy for select grade 1 disease, with excellent local control and toxicity rates. Further work is needed to define optimal timing and fractionation. 

### Grade 2

Several retrospective studies defined that recurrence risk after GTR for Grade 2 meningioma is between 40 and 60%, and recurrence typically occurs within 5 years after surgery [34]. These also helped establish retrospectively that recurrence risks after GTR + RT were significantly lower, between 0 and 30%. This has driven prospective investigation of escalated therapy with postoperative radiotherapy. RTOG0539 studied these tumors prospectively on their intermediate risk arm, which received 54 Gy in 30 fractions and found a 5-year PFS of 84%, with no G3-4 toxicities [35]. EORTC 22042–26042 was a phase II cohort study that took these tumors and treated them to 60 Gy in 30 fractions, resulting in 89% 3-year PFS with grade 3 toxicity rates of 14%, which was higher than observed toxicity on RTOG-0539 [36].

Recent studies and meta-analyses support the benefit of postoperative RT. A multi-institutional propensity score-matched study of 518 patients found that 5-year PFS was significantly better (85% versus 65%) in patients who received RT, and a LC benefit was found in low-, intermediate-, and high-risk groups, which were defined based on surgical extent, Ki-67 index, and tumor size [37]. A single-institution retrospective study identified 230 patients with resected grade 2 disease; receipt of RT was found to be associated with a lower risk of recurrence despite higher-risk disease [38]. Additionally, patients who were irradiated as salvage therapy were found to have shorter post-RT PFS than those who were treated in the adjuvant setting (19 versus 64 months). Three systematic reviews and meta-analyses have been published recently that demonstrate similar results. One identified 2,008 patients and found better LC (82% versus 71%) and PFS with adjuvant RT without an observed OS benefit [39]; another identified 3,078 patients and found improved LC (HR = 0.7), PFS (HR = 0.69) and OS (HR = 0.55) [40]; and another identified 2,904 patients and found reduction of LR with an OR of 0.5, improvement of PFS, and no significant difference in OS with a tolerable toxicity profile [41].

Prospective, randomized data is needed to solidify the role of RT in this scenario. This work is underway with the two international randomized trials, ROAM/EORTC-1308/TROG 15.02 and NRG-BN003 [12, 42]. These studies are both comparing early adjuvant fractionated RT with active surveillance after GTR for grade 2 disease. ROAM has completed accrual and BN003 is still accruing at time of writing. These anticipated publications will further define the role of adjuvant RT in this setting.

Overall, the accumulation of evidence for adjuvant RT in this setting has led to guideline recommendations for consideration of RT after GTR for grade 2 meningioma [3].

Briefly, although radiosurgery has excellent outcomes for grade 1 disease, the outcomes for grade 2/3 disease are uncertain. One recent multi-institutional retrospective study analyzed 233 patients with grade 2 disease, many with prior surgery or radiation, who received SRS [43]. Median dose was 15 Gy, and 3-year PFS was 54%. Another single-institution retrospective study of 48 patients found a 5-year PFS of 46% [44]. An earlier systematic review of 19 series identified 647 patients with grade 2 or 3 disease treated with SRS; median 5-year PFS was 59% in grade 2 tumors and 13% for grade 3 tumors [45]. Careful patient selection is required for SRS in grade 2 or 3 disease.

### Grade 3, dose escalation, and particle therapy

Grade 3 disease is managed uniformly with surgery followed by RT [3]. RTOG 0539 studied these tumors prospectively on their high-risk arm, which received 60 Gy in 30 fractions and found a 3-year PFS of 59% [46]. These poor outcomes have prompted further investigation of strategies to refine the diagnosis and management of this disease. Patterns of failure have been studied, and these largely take place in-field after fractionated RT [47–49]. Based on this, and existing evidence for dose thresholds that improve LC [50], escalation beyond historic doses of 60 Gy is one natural avenue to try and improve LC.

Recently, Sahgal and colleagues provided pivotal evidence that dose escalation improves local control for atypical and anaplastic meningiomas [51]. They retrospectively compared dose-escalated RT (≥ 66 Gy) versus standard dose (< 66 Gy) in 118 (94% grade 2; 6% grade 3) meningiomas. Dose escalation was associated with better PFS **(**3-/5-yr PFS 78.9/64.6% vs. 57.2/40.8%), and multivariable analyses confirmed dose escalation improved PFS (HR 0.54) and reduced local failure (HR 0.48). Symptomatic radionecrosis occurred in 5.9% overall with no difference in toxicity between cohorts. Overall, this suggests escalation to ≥ 66 Gy can improve control with acceptable toxicity.

Other retrospective single-institution studies have found similar results. One cohort of 158 patients with resected grade 2 disease was identified, in which higher RT dose (above EQD2 57.42 Gy) was significantly associated with improved LC, PFS, and OS; and a dose-dependent effect on LC of about 12%/Gy was demonstrated [52]. Another single-institution retrospective cohort study found excellent results using a boost to GTV to 66 Gy, with 5-year PFS of 67.5% in grade 3 disease and 90.4% 5-year LC in grade 2 disease [49]. Systematic reviews of dose escalation, although the studies included are markedly heterogeneous, have shown that RT dose and modern techniques were associated with improved PFS in grade 2 and 3 disease [53–55].

Combined approaches using photon-particle and conventional-SRS treatments have also been used to escalate therapy. A classic Phase I/II study dose-escalated treatment with a mixed photon-proton approach to 68–72 Gy, which had excellent control and low toxicity in a small group of patients [56]. A subsequent randomized trial examined 55.8 Gy versus 63 Gy using this approach for grade 1 disease which did not show any significant control differences at long-term follow-up [57]. Conventional RT with an SRS boost to gross disease was examined in a single-center series of high-risk grade 2 tumors, which was feasible and safe, and detected a signal for efficacy after STR [58].

Historically, proton therapy has been utilized for meningiomas due to the potential for decreased toxicity, particularly in the skull base. A retrospective cohort study of 200 patients who underwent proton therapy after STR or biopsy (66%) for grade 1–3 meningiomas (70% grade 1, 27.5% grade 2, 2.5% grade 3) located primarily at the skull base found excellent local control (5-year LC of 97.5% for grade 1, 77.8% for grade 2/3) [59]. Acute grade 3 + toxicities were uncommon (1%), grade 3 + late toxicities occurred in 12% of patients, and patient QoL was preserved. Overall, this toxicity was comparable to the high-risk arm of RTOG0539 (grade 3 + rate of 13%).

A systematic review identified 240 patients with grade 2 or grade 3 disease who underwent particle therapy, and suggested disease control, survival, and toxicity benefit compared to photon therapy [60]. The studies included in this review were markedly heterogeneous, however, and it is difficult to draw conclusions from existing particle therapy data.

The prospective phase 2 MARCIE trial investigated combined modality photon-carbon ion therapy with an 18 Gy boost in 6 fractions delivered with carbon ions following 50 Gy in 25 fractions with photons for grade 2 meningiomas following STR [61]. 3-year PFS was estimated to be 80.3%. Radiation necrosis occurred in 3/33 patients, one of whom died after resection.

The question of benefit to particle therapy has motivated the design of prospective trials. NCT0293990 is investigating increased-dose proton therapy with a primary outcome of safety by identifying dose-limiting toxicity [62]. PANAMA is a multi-arm non-randomized study that is escalating gross disease to 68 Gy (Grade 2 STR) or 72 Gy (Grade 3 STR) using combined modality therapy, while treating GTR tumors to a standard 60 Gy dose [63]. COG-PROTON-01 is randomizing patients to proton or photon therapy for grade 1 tumors in the cavernous sinus, with a primary endpoint of composite long-term function and neurocognition [64]. Ongoing radiotherapy trials in the non-salvage setting are summarized in Table 1.

Overall, these studies show that escalation of dose is a promising avenue for increased disease control. With the advent of proton therapy and DOTATATE imaging, the integration of these into treatment planning to escalate dose and improve local control in high-grade disease is a subject of current interest. The relatively low reported alpha/beta ratio of meningiomas (3.3–3.7 in one meta-analysis) [65] also suggests that hypofractionation could result in improved local control, with the possibility of combined modality or fractionation approaches to respect OAR dose limits and address marginal failure risk.

## Salvage Therapy

Recurrent meningiomas pose significant challenges, as grade 2/3 tumors frequently recur and retreatment with surgery and radiation is limited. Additionally, there is limited evidence to support the use of any therapeutic strategy for recurrence, and thus treatment is heterogeneous and depends heavily on institutional preference, access to technology, and clinical trial availability.

Reirradiation is a frequent strategy for recurrent meningioma. Several recent single-institution retrospective studies have been performed recently summarizing their experience with particle therapy in this setting. One identified 42 patients who received a median of 51 Gy in 19 fractions, which resulted in a 2-year PFS of 56.5%, with no observed grade 4 or 5 toxicities [66]. Another found 16 patients who underwent proton therapy, and found a 2-year PFS of 43% with late grade 3 + toxicity rates of 31% [67]. Another study of 32 patients found a 2-year PFS of 74.5%, in addition to low rates of grade 3 + toxicity [68]. A larger multi-institutional study that included 181 patients who underwent proton or photon therapy (the majority SRS or FSRT, with a median PTV volume of 6.5 cc) found a 3-year PFS of 52%, with no grade 3 + late toxicity and only 2% acute grade 3 + toxicity [69].

The use of pulsed-reduced dose rate (PRDR) therapy for brain tumors has been of interest recently for reirradiation, as NRG-CC017 will study this technique for gliomas. PRDR utilizes temporally separated pulses of RT to take advantage of sublethal damage repair in normal tissues, low-dose hyper-radiosensitivity, and cell-cycle redistribution to exert biologic tumor effect while protecting OARs. Single-institution studies treating gliomas with large-volume reirradiation PRDR have been published with favorable outcomes and toxicity [70, 71]. A small published study at our institution found excellent toxicity and control with large-volume meningioma PRDR reirradiation [72], a recent unpublished update of which included 26 patients and found a median PFS of 27 months and grade 3 + toxicity rates of 15%.

Historically, systemic therapies such as hydroxyurea, interferon-α, or somatostatin analogues have yielded limited benefit, primarily with stabilization of disease and 6-month PFS rates of 15–50% [73–75]. Recent years have seen substantial growth in clinical trials exploring targeted and immune-based approaches.

VEGF inhibitors have shown modest activity, stabilizing disease in subsets of patients. Retrospective and prospective examination of bevacizumab has shown promising 6-month PFS rates ranging from 44% to 85% [76, 77]. Sunitinib has been examined prospectively and had a 6-month PFS of 42% with high rates of grade 3 + toxicity [78]. mTOR pathway inhibitors are under investigation, as a recent trial combining everolimus and octreotide had 6-month PFS of 55% with substantial reductions in tumor growth rate [79]. Checkpoint inhibitors, including nivolumab and pembrolizumab, are being tested in high-grade and recurrent meningiomas, with generally low 6-month PFS rates [80, 81].

Peptide receptor radionuclide therapy (PRRT), leveraging DOTATATE uptake, has generated enthusiasm as a salvage modality. A meta-analysis summarized early trials of SSTR PRRT for meningioma, reporting 6-month PFS of 61% and 12-month PFS of 53% [82]. Stratification by grade showed 6-month PFS rates of 94%, 48%, and 0% for grade 1, 2, and 3 disease, respectively. A more recent prospective phase II trial reported 6-month PFS of 77.8% in a grade 2/3 cohort, with grade 3 + toxicity of 46% [83]. A recent retrospective study reviewed 37 patients and found median PFS of 15 months, although a substantial proportion of these patients were grade 1 [84]. Another small prospective study of primarily grade 2 patients found a 6-month PFS of 50% [85]. Based on these studies, PRRT seems to offer more promising disease control than other systemic therapies, although the optimal use and dosing of PRRT remains to be determined. Randomized trials through the EORTC and RTOG are underway [86, 87], which will further delineate the clinical utility of this modality. These and other ongoing trials in the salvage setting are summarized in Table 2.

Overall, salvage outcomes remain suboptimal, underscoring the need for continued clinical trial enrollment. The integration of molecular and imaging signatures into the choice of salvage therapy is critical to identify patients most likely to benefit from specific therapies.

## Conclusions

The convergence of molecular biology, functional imaging, and advanced therapeutics is reshaping the meningioma field. Classification schemes are moving beyond histology towards integrated molecular–imaging taxonomies. DOTATATE-PET and radiomics are providing real-time biological insights that augment surgical and radiotherapeutic planning. Clinical trials are testing agents rationally matched to molecular subgroups.

Meningioma care is rapidly becoming personalized. The validation and integration of molecular and imaging biomarkers, will improve prognostication and therapeutic decision-making. This will result in establishment of a unified molecular classification and the routine use of functional imaging in surgical and RT workflows. These advances promise more effective and less morbid treatments, particularly for aggressive or refractory disease.

**Table 1:** Selected Ongoing Clinical Trials in the Intact Setting

Trial ID	Intervention	Population	Notes
NRG-BN003 (NCT03180268)	Adjuvant RT vs. Observation after GTR	WHO grade II (atypical) meningioma, GTR (Simpson I–III)	Phase III, randomized; PFS primary endpoint; Active multi-center
ROAM / EORTC-1308 / TROG 15.02 (ISRCTN71502099)	Early adjuvant RT (60 Gy/30) vs. Active Monitoring	Post-GTR atypical (grade II) meningioma	Phase III, randomized; Closed to accrual; results pending publication
NCT02693990	Dose-escalated Intensity Modulated Proton Therapy (IMPT)	High-risk grade II–III; after STR/biopsy or high-risk features	Phase I/II; IMPT dose escalation; safety/efficacy endpoints
NCT06937268	DOTATATE PET-guided RT planning (PET/MRI integration)	Intracranial meningioma candidates for postoperative RT	Prospective study of PET-guided target delineation

**Table 2:** Selected Ongoing Clinical Trials in the Salvage Setting

Trial ID	Intervention	Population	Notes
NCT03279692	Pembrolizumab (PD-1 inhibitor)	Recurrent/progressive grade II–III meningioma	Phase II; PFS-6 primary endpoint; results published; signal of benefit in subset
NCT04659811	SRS + Pembrolizumab	Recurrent meningioma eligible for SRS	Phase II; combination radio-immunotherapy
NCT03971461	177Lu-DOTATATE (PRRT)	Progressive WHO I–III or residual high-risk, SSTR-positive	Phase II, single-arm; multicenter
LUMEN-1 (NCT06326190)	[177Lu]Lu-DOTATATE vs. Best Investigator’s Choice (control)	Refractory/progressive meningioma (SSTR-positive)	Randomized Phase II; EORTC-led; launched 2025
Alliance A071401 (NCT02523014)	Molecularly matched therapy (e.g., capivasertib; vismodegib; FAK inhibitor)	Progressive meningioma with pathway-specific alterations	Phase II; capivasertib arm reported open at select sites
NCT02847559	Optune (TTFields) + Bevacizumab	Recurrent/progressive grade II–III meningioma	Phase II; device + anti-VEGF
NCI-2016-00504	Nivolumab ± Ipilimumab	Recurrent grade I–III meningioma	Phase II; immune checkpoint blockade

## Key References

- JZ W, AP L, DR R, F S, KM W, R G. Meningioma: International Consortium on Meningiomas consensus review on scientific advances and treatment paradigms for clinicians, researchers, and patients. Neuro-Oncology. 2024;26(10):1742−80. 10.1093/neuonc/noae082.
  - ○ Recent consensus review.
- Gui C, Wang JZ, Patil V, Landry AP, Singh O, Castelo-Branco P, et al. Analysis of TERT association with clinical outcome in meningiomas: a multi-institutional cohort study. Lancet Oncol. 2025;26(9):1191−203. 10.1016/S1470-2045(25)00267-0.
  - ○ Recent molecular classification advance.
- Groff KJ, Patel RV, Feng Y, Ghosh HS, Millares Chavez MA, O’Brien J, et al. The effect of TERT promoter mutation on predicting meningioma outcomes: a multi-institutional cohort analysis. Lancet Oncol. 2025;26(9):1178−90. 10.1016/S1470-2045(25)00422-X.
  - ○ Recent molecular classification advance.
- JZ W, C G, DR R. Molecular classification to refine surgical and radiotherapy care for meningioma. Nature Medicine. 2024. 10.1038/s41591-024-03167-4.
  - ○ Recent molecular classification advance.
- Landry AP, Wang JZ, Patil V, Liu J, Gui C, Ellenbogen Y, et al. Chromosome 1p Loss and 1q Gain for Grading of Meningioma. JAMA Oncol. 2025;11(6):644−9. 10.1001/jamaoncol.2025.0329.
  - ○ Recent molecular classification advance.
- HK P, TJC W, DR R. 68Ga-DOTATATE PET-based radiation contouring creates more precise radiation volumes for meningioma patients. International Journal of Radiation Oncology Biology Physics. 2022;112(2):E63−E4. 10.1016/j.ijrobp.2022.04.033.
  - ○ Largest study to show radiotherapy volume refinement with DOTATATE-PET.
- J I, SJ C, A H. DOTATATE PET/MRI–guided radiosurgical planning and response assessment in meningiomas. Neuro-Oncology. 2024;26(8):1526−35. 10.1093/neuonc/noae067.
  - ○ First study to assess response assessment by DOTATAE-PET after RT.
- Albert NL, Preusser M, Traub-Weidinger T, Tolboom N, Law I, Palmer JD, etal. Joint EANM/EANO/RANO/SNMMI practice guideline/procedure standards for diagnostics and therapy (theranostics) of meningiomas using radiolabeled somatostatin receptor ligands: version 1.0. Eur J Nucl Med Mol Imaging.2024;51(12):3662−79. 10.1007/s00259-024-06783-x.
  - ○ New expert practice guideline for diagnostics.
- HK P, DR R, TJC W, JD P. Consensus radiation treatment planning guidelines utilizing 68Ga-DOTATATE PET/CT for resected meningiomas. International Journal of Radiation Oncology Biology Physics. 2025;122(1):150−8. 10.1016/j.ijrobp.2024.11.023.
  - ○ New consensus treatment planning guidelines incorporating PET.
- Rogers CL, Pugh SL, Vogelbaum MA, Perry A, Ashby LS, Modi JM, et al. Low-risk meningioma: Initial outcomes from NRG Oncology/RTOG 0539. Neuro Oncol. 2023;25(1):137−45.
  - ○ Largest prospective radiotherapy meningioma trial results.
- Zeng KL, Soliman H, Myrehaug S, Tseng CL, Detsky JM, Chen HM, et al. Dose-Escalated Radiation Therapy Is Associated With Improved Outcomes for High-Grade Meningioma. Int J Radiat Oncol Biol Phys. 2024;118(3):662−71. 10.1016/j.ijrobp.2023.09.026.
  - ○ Recent evidence supporting dose escalation.
- Desideri I, Morelli I, Banini M, Greto D, Visani L, Nozzoli F, et al. Re-irradiation for recurrent intracranial meningiomas: Analysis of clinical outcomes and prognostic factors. Radiother Oncol. 2024;195:110271. 10.1016/j.radonc.2024.110271.
  - ○ Largest reirradiation series.

## Data Availability

No datasets were generated or analysed during the current study.

## References

[1] JZ W, DR APL, KM RFS, Meningioma WRG. International Consortium on Meningiomas consensus review on scientific advances and treatment paradigms for clinicians, researchers, and patients. Neurooncology. 2024;26(10):1742–80. 10.1093/neuonc/noae082. 10.1093/neuonc/noae082 PMC11449035 38695575

[2] DN L. The 2021 WHO classification of tumors of the central nervous system: a summary. Neurooncology. 2021;23(8):1231–51. 10.1093/neuonc/noab106. 10.1093/neuonc/noab106 PMC8328013 34185076

[3] Nabors LB, Portnow J, Ahluwalia M, Baehring J, Brem H, Brem S, et al. Central Nervous System Cancers, Version 3.2020, NCCN Clinical Practice Guidelines in Oncology. J Natl Compr Canc Netw. 2020;18(11):1537–70. 10.6004/jnccn.2020.0052. 33152694 10.6004/jnccn.2020.0052

[4] Gui C, Wang JZ, Patil V, Landry AP, Singh O, Castelo-Branco P, et al. Analysis of TERT association with clinical outcome in meningiomas: a multi-institutional cohort study. Lancet Oncol. 2025;26(9):1191–203. 10.1016/S1470-2045(25)00267-0. 40907516 10.1016/S1470-2045(25)00267-0

[5] Groff KJ, Patel RV, Feng Y, Ghosh HS, Millares Chavez MA, O’Brien J, et al. The effect of TERT promoter mutation on predicting meningioma outcomes: a multi-institutional cohort analysis. Lancet Oncol. 2025;26(9):1178–90. 10.1016/S1470-2045(25)00422-X. 40907515 10.1016/S1470-2045(25)00422-X PMC13090858

[6] F S, K S, I M. DNA methylation-based classification and grading system for meningioma. Lancet Oncol. 2017;18(5):682–94. https://pubmed.ncbi.nlm.nih.gov/28314689/28314689 10.1016/S1470-2045(17)30155-9

[7] F N YM. Meningioma classification by DNA methylation profiling shows clinically relevant groups. Nature. 2021;597(7874):119–25. 10.1038/s41586-021-03850-3. 34433969 10.1038/s41586-021-03850-3 PMC11604310

[8] JZ W, C G. Molecular classification to refine surgical and radiotherapy care for meningioma. Nat Med. 2024. 10.1038/s41591-024-03167-4. 10.1038/s41591-024-03167-4 PMC11564112 39169220

[9] MW Y. A 34-gene expression signature predicts benefit from radiotherapy in meningioma. Neurooncology. 2022. 10.1093/neuonc/noac089.

[10] Landry AP, Wang JZ, Patil V, Liu J, Gui C, Ellenbogen Y, et al. Chromosome 1p Loss and 1q Gain for Grading of Meningioma. JAMA Oncol. 2025;11(6):644–9. 10.1001/jamaoncol.2025.0329. 40178835 10.1001/jamaoncol.2025.0329 PMC11969356

[11] MD RGPS EANO guideline on the diagnosis and management of meningiomas. Neurooncology. 2021;23(11):1821–34. . 10.1093/neuonc/noab150. 10.1093/neuonc/noab150 PMC8563316 34181733

[12] 2017. https://clinicaltrials.gov/study/NCT03180268

[13] TA B. Intracranial meningioma: A review of recent and emerging data on the utility of PET imaging. J Neuroimaging. 2024. 10.1111/jon.13570. 10.1111/jon.13227 39113129

[14] W R, VM S. 68Ga-DOTATATE uptake in meningioma correlates with SSTR2 expression and enables accurate delineation. J Nucl Med. 2015;56(3):347–53. 10.2967/jnumed.114.149120. 25635133 10.2967/jnumed.114.149120

[15] WG K, NL J. Improved detection of transosseous meningiomas using 68Ga-DOTATATE PET/CT compared with contrast-enhanced MRI. J Nucl Med. 2017;58(10):1580–5. 10.2967/jnumed.116.189365. 28450556 10.2967/jnumed.117.191932

[16] R G, N W, U M-L. Target volume delineation of skull base meningiomas with 68Ga-DOTATOC PET/CT: Impact on radiotherapy planning. Int J Radiat Oncol Biol Phys. 2013;85(1):68–73. 10.1016/j.ijrobp.2012.03.043. 22575489 10.1016/j.ijrobp.2012.03.021

[17] S M-Z, A Z-dB MH. Target definition of meningiomas by PET with 68Ga-DOTATOC for stereotactic radiotherapy. Int J Radiat Oncol Biol Phys. 2006;65(3):911–4. 10.1016/j.ijrobp.2006.01.036. 10.1016/j.ijrobp.2005.12.006 16488553

[18] HK P, TJC W. 68Ga-DOTATATE PET-based radiation contouring creates more precise radiation volumes for meningioma patients. Int J Radiat Oncol Biol Phys. 2022;112(2):E63–4. 10.1016/j.ijrobp.2022.04.033. 10.1016/j.ijrobp.2022.04.009 35460804

[19] ES K, SE C. DOTATATE PET/CT for radiation planning and response assessment in meningioma. Radiat Oncol. 2021;16(1):191. 10.1186/s13014-021-01875-6.34583727

[20] SH K, HS L, SW O. Evaluating diagnostic accuracy of quantitative 68Ga-DOTATATE PET/MRI analysis in meningioma. Sci Rep. 2022;12(1):9165. 10.1038/s41598-022-13467-9.35655078

[21] KA K. 68Ga-DOTATATE PET enables precise target volume delineation and improves outcomes in low-grade meningioma. Eur J Nucl Med Mol Imaging. 2020;47(1):86–94. 10.1007/s00259-019-04539-0.

[22] H M, A M, N T. Integration of DOTATATE PET with fractionated stereotactic radiotherapy for meningioma: disease control and functional outcomes. Neuro-Oncology Adv. 2021;3(1):vdab024. 10.1093/noajnl/vdab024.

[23] SJ JI. DOTATATE PET/MRI–guided radiosurgical planning and response assessment in meningiomas. Neurooncology. 2024;26(8):1526–35. 10.1093/neuonc/noae067. 10.1093/neuonc/noae067 PMC11300004 38553990

[24] Albert NL, Preusser M, Traub-Weidinger T, Tolboom N, Law I, Palmer JD, et al. Joint EANM/EANO/RANO/SNMMI practice guideline/procedure standards for diagnostics and therapy (theranostics) of meningiomas using radiolabeled somatostatin receptor ligands: version 1.0. Eur J Nucl Med Mol Imaging. 2024;51(12):3662–79. 10.1007/s00259-024-06783-x. 38898354 10.1007/s00259-024-06783-x PMC11445317

[25] HK P, DR R, TJC W. Consensus radiation treatment planning guidelines utilizing 68Ga-DOTATATE PET/CT for resected meningiomas. Int J Radiat Oncol Biol Phys. 2025;122(1):150–8. 10.1016/j.ijrobp.2024.11.023. 39701546 10.1016/j.ijrobp.2024.12.003

[26] Rogers CL, Pugh SL, Vogelbaum MA, Perry A, Ashby LS, Modi JM, et al. Low-risk meningioma: Initial outcomes from NRG Oncology/RTOG 0539. Neuro Oncol. 2023;25(1):137–45. 10.1093/neuonc/noac137. 35657335 10.1093/neuonc/noac137 PMC9825319

[27] WC C, ME D. Radiotherapy and radiosurgery for meningiomas. Neuro-Oncology Adv. 2023;5(1):vdad125. 10.1093/noajnl/vdad125. 10.1093/noajnl/vdac088 PMC10498143 37711972

[28] MT M. Single- and multi-fraction stereotactic radiosurgery dose tolerances of the optic pathways. Int J Radiat Oncol Biol Phys. 2021;110(1):87–99. 10.1016/j.ijrobp.2018.01.053. 29534899 10.1016/j.ijrobp.2018.01.053 PMC9479557

[29] Vaishnav YJ, Singh R, Didwania P, Lehrer EJ, Bakaeva T, Harris TJ, et al. Radiotherapy and Radiosurgery in the Management of Optic Nerve Sheath Meningiomas: An International Systematic Review and Meta-Analysis of Twenty Studies. World Neurosurg. 2022;164:e929–44. 10.1016/j.wneu.2022.05.064. 35609728 10.1016/j.wneu.2022.05.064

[30] Chen HY, Chuang CC, Chen HC, Wei KC, Chang CN, Liu ZH, et al. Clinical outcomes of fractionated stereotactic radiosurgery in treating perioptic meningiomas and schwannomas: A single-institutional experience. J Clin Neurosci. 2020;81:409–15. 10.1016/j.jocn.2020.09.058. 33222952 10.1016/j.jocn.2020.09.058

[31] Bunevicius A, Anand RK, Suleiman M, Nabeel AM, Reda WA, Tawadros SR, et al. Stereotactic Radiosurgery for Perioptic Meningiomas: An International, Multicenter Study. Neurosurgery. 2021;88(4):828–37. 10.1093/neuros/nyaa544. 33475718 10.1093/neuros/nyaa544 PMC8517876

[32] Milano MT, Grimm J, Soltys SG, Yorke E, Moiseenko V, Tome WA, et al. Single- and Multi-Fraction Stereotactic Radiosurgery Dose Tolerances of the Optic Pathways. Int J Radiat Oncol Biol Phys. 2021;110(1):87–99. 10.1016/j.ijrobp.2018.01.053. 29534899 10.1016/j.ijrobp.2018.01.053 PMC9479557

[33] Marchetti M, Sahgal A, De Salles AAF, Levivier M, Ma L, Paddick I, et al. Stereotactic Radiosurgery for Intracranial Noncavernous Sinus Benign Meningioma: International Stereotactic Radiosurgery Society Systematic Review, Meta-Analysis and Practice Guideline. Neurosurgery. 2020;87(5):879–90. 10.1093/neuros/nyaa169. 32463867 10.1093/neuros/nyaa169 PMC7566438

[34] Hasan S, Young M, Albert T, Shah AH, Okoye C, Bregy A, et al. The role of adjuvant radiotherapy after gross total resection of atypical meningiomas. World Neurosurg. 2015;83(5):808–15. 10.1016/j.wneu.2014.12.037. 25535067 10.1016/j.wneu.2014.12.037

[35] CL R, WA PZ. Intermediate-risk meningioma: Initial outcomes from NRG Oncology/RTOG 0539. Int J Radiat Oncol Biol Phys. 2018;103(3):861–8. 10.1016/j.ijrobp.2018.11.054.30419305

[36] C RM. EORTC 22042–26042: Adjuvant radiotherapy for atypical meningioma after gross total resection – phase II results. Radiother Oncol. 2020;145:155–61. 10.1016/j.radonc.2019.12.002.

[37] HK B, JH C. Adjuvant radiotherapy versus surveillance for grade 2 meningiomas: a multicenter propensity score–matched study. Front Oncol. 2022;12:877244. 10.3389/fonc.2022.877244. 35847889 10.3389/fonc.2022.877244 PMC9283569

[38] JW JL, CK K. Adjuvant radiotherapy after gross total resection for atypical meningioma: a single-institution experience. J Neurooncol. 2021;154(1):55–63. 10.1007/s11060-021-03724-5.

[39] H Q, Z Y, L J. Meta-analysis of adjuvant radiotherapy after gross total resection of atypical meningioma. Front Oncol. 2021;11:702732. 10.3389/fonc.2021.702732.

[40] Y S, M Z, J C. Adjuvant radiotherapy improves outcomes in atypical meningioma: a systematic review and meta-analysis. Acta Neurochir. 2022;164(5):1261–71. 10.1007/s00701-021-05170-6

[41] SW C, KM K, MS K. Adjuvant radiotherapy versus observation following gross total resection for atypical meningioma: a systematic review and meta-analysis. Radiat Oncol. 2021;16(1):34. 10.1186/s13014-021-01759-9. 33596974 10.1186/s13014-021-01759-9 PMC7890913

[42] Jenkinson MD, Javadpour M, Haylock BJ, Young B, Gillard H, Vinten J, et al. The ROAM/EORTC-1308 trial: Radiation versus Observation following surgical resection of Atypical Meningioma: study protocol for a randomised controlled trial. Trials. 2015;16:519. 10.1186/s13063-015-1040-3. 26576533 10.1186/s13063-015-1040-3 PMC4650615

[43] Kowalchuk RO, Shepard MJ, Sheehan K, Sheehan D, Faramand A, Niranjan A, et al. Treatment of WHO Grade 2 Meningiomas With Stereotactic Radiosurgery: Identification of an Optimal Group for SRS Using RPA. Int J Radiat Oncol Biol Phys. 2021;110(3):804–14. 10.1016/j.ijrobp.2021.01.048. 33548341 10.1016/j.ijrobp.2021.01.048

[44] Helis CA, Hughes RT, Cramer CK, Tatter SB, Laxton AW, Bourland JD, et al. Stereotactic Radiosurgery for Atypical and Anaplastic Meningiomas. World Neurosurg. 2020;144:e53–61. 10.1016/j.wneu.2020.07.211. 32758657 10.1016/j.wneu.2020.07.211

[45] Ding D, Starke RM, Hantzmon J, Yen CP, Williams BJ, Sheehan JP. The role of radiosurgery in the management of WHO Grade II and III intracranial meningiomas. Neurosurg Focus. 2013;35(6):E16. 10.3171/2013.9.FOCUS13364. 24289124 10.3171/2013.9.FOCUS13364

[46] CL R. High-risk meningioma: Initial outcomes from NRG Oncology/RTOG 0539. Int J Radiat Oncol Biol Phys. 2019;104(5):1046–54. 10.1016/j.ijrobp.2019.04.011. 10.1016/j.ijrobp.2019.11.028 PMC7117785 31786276

[47] Adeberg S, Hartmann C, Welzel T, Rieken S, Habermehl D, von Deimling A, et al. Long-term outcome after radiotherapy in patients with atypical and malignant meningiomas–clinical results in 85 patients treated in a single institution leading to optimized guidelines for early radiation therapy. Int J Radiat Oncol Biol Phys. 2012;83(3):859–64. 10.1016/j.ijrobp.2011.08.010. 22137023 10.1016/j.ijrobp.2011.08.010

[48] Susko MS, Chen WC, Vasudevan HN, Magill ST, Lucas CG, Oberheim Bush NA, et al. Letter: Patterns of Intermediate- and High-Risk Meningioma Recurrence After Treatment With Postoperative External Beam Radiotherapy. Neurosurgery. 2021;89(1):E99–101. 10.1093/neuros/nyab143. 33887769 10.1093/neuros/nyab143 PMC8203419

[49] Lee JJB, Lee J, Yoon HI, Kim SH, Cho J, Lee KS, et al. Analysis of patterns of failure and appraisal of postoperative radiation field for grade II-III meningioma. J Neurooncol. 2019;144(2):333–41. 10.1007/s11060-019-03232-w. 31278690 10.1007/s11060-019-03232-w

[50] Goldsmith BJ, Wara WM, Wilson CB, Larson DA. Postoperative irradiation for subtotally resected meningiomas. A retrospective analysis of 140 patients treated from 1967 to 1990. J Neurosurg. 1994;80(2):195–201. 10.3171/jns.1994.80.2.0195. 8283256 10.3171/jns.1994.80.2.0195

[51] Zeng KL, Soliman H, Myrehaug S, Tseng CL, Detsky JM, Chen HM, et al. Dose-Escalated Radiation Therapy Is Associated With Improved Outcomes for High-Grade Meningioma. Int J Radiat Oncol Biol Phys. 2024;118(3):662–71. 10.1016/j.ijrobp.2023.09.026. 37793575 10.1016/j.ijrobp.2023.09.026

[52] Kim D, Chang WI, Byun HK, Kim IA, Cho J, Lee JH, et al. Dose-response relationship in patients with newly diagnosed atypical meningioma treated with adjuvant radiotherapy. J Neurooncol. 2023;161(2):329–37. 10.1007/s11060-022-04206-1. 36469188 10.1007/s11060-022-04206-1

[53] Jahanbakhshi A, Najafi M, Gomar M, Ciammella P, Ruggieri MP, Iotti C, et al. Radiosurgery in Grade II and III Meningiomas: A Systematic Review and Meta-Analysis. J Pers Med. 2024;14(8). 10.3390/jpm14080802. 10.3390/jpm14080802 PMC11355310 39201994

[54] Ehret F, El Baya L, Erridge SC, Bussiere M, Verhoeff JJC, Niyazi M, et al. Radiation Therapy for Meningiomas - Where Do We Stand and What’s on the Horizon? Int J Radiat Oncol Biol Phys. 2025;121(3):599–612. 10.1016/j.ijrobp.2024.10.034. 39476990 10.1016/j.ijrobp.2024.10.034

[55] Gaito S, Goyal L, Rieu R, France A, Burnet NG, Barker C, et al. Radiotherapy intensification for atypical and malignant meningiomas: A systematic review. Neurooncol Pract. 2024;11(2):115–24. 10.1093/nop/npad077. 38496911 10.1093/nop/npad077 PMC10940825

[56] Chan AW, Bernstein KD, Adams JA, Parambi RJ, Loeffler JS. Dose escalation with proton radiation therapy for high-grade meningiomas. Technol Cancer Res Treat. 2012;11(6):607–14. 10.7785/tcrt.2012.500267. 22712601 10.7785/tcrt.2012.500267

[57] Sanford NN, Yeap BY, Larvie M, Daartz J, Munzenrider JE, Liebsch NJ, et al. Prospective, Randomized Study of Radiation Dose Escalation With Combined Proton-Photon Therapy for Benign Meningiomas. Int J Radiat Oncol Biol Phys. 2017;99(4):787–96. 10.1016/j.ijrobp.2017.07.008. 28865924 10.1016/j.ijrobp.2017.07.008 PMC5654667

[58] Calafiore RL, Helis CA, Marcet P, Smith E, Ramsey B, Pacholke H, et al. Fractionated Radiotherapy With Stereotactic Radiosurgery Boost Controls Gross Disease in Grade 2 Meningioma. World Neurosurg. 2025;194:123429. 10.1016/j.wneu.2024.11.012. 39579930 10.1016/j.wneu.2024.11.012

[59] Krcek R, Leiser D, Garcia-Marqueta M, Bolsi A, Weber DC. Long Term Outcome and Quality of Life of Intracranial Meningioma Patients Treated with Pencil Beam Scanning Proton Therapy. Cancers (Basel). 2023;15(12). 10.3390/cancers15123099. 10.3390/cancers15123099 PMC10296362 37370709

[60] EB AW. S, JP S. Particle therapy for high-grade meningiomas: a systematic review. Neurosurgical Focus. 2019;46(6):E10. 10.3171/2019.3.Focus1975.

[61] A S, A A, A S. Carbon ion boost following photon radiotherapy for atypical meningioma (MARCIE): Phase II trial. Radiat Oncol. 2018;13(1):4. . 10.1186/s13014-017-0948-929325590

[62] 2016. https://clinicaltrials.gov/study/NCT02693990

[63] 2016. https://clinicaltrials.gov/study/NCT02978677

[64] Lesueur P, Clarisse B, Lequesne J, Licaj I, Feuvret L, Stefan D, et al. Proton therapy versus conventional radiotherapy for the treatment of cavernous sinus benign meningioma, a randomized controlled phase III study protocol (COG-PROTON-01). BMC Cancer. 2024;24(1):1594. 10.1186/s12885-024-13353-9. 39736544 10.1186/s12885-024-13353-9 PMC11687058

[65] Vernimmen FJ, Slabbert JP. Assessment of the alpha/beta ratios for arteriovenous malformations, meningiomas, acoustic neuromas, and the optic chiasma. Int J Radiat Biol. 2010;86(6):486–98. 10.3109/09553001003667982. 20470198 10.3109/09553001003667982

[66] RA E-S AA. Particle therapy reirradiation for recurrent intracranial meningioma: outcomes and toxicity. Radiat Oncol. 2018;13(1):86. 10.1186/s13014-018-1026-0. 29739417 10.1186/s13014-018-1026-x PMC5941671

[67] Imber BS, Neal B, Casey DL, Darwish H, Lin AL, Cahlon O, et al. Clinical Outcomes of Recurrent Intracranial Meningiomas Treated with Proton Beam Reirradiation. Int J Part Ther. 2019;5(4):11–22. 10.14338/IJPT-18-00045.1. 31773037 10.14338/IJPT-18-00045.1 PMC6871625

[68] Scartoni D, Giacomelli I, Pertile R, Vennarini S, Feraco P, Picori L, et al. Proton therapy re-irradiation provides promising clinical results in recurrent brain meningioma. Acta Oncol. 2023;62(9):1096–101. 10.1080/0284186X.2023.2241994. 37526998 10.1080/0284186X.2023.2241994

[69] Desideri I, Morelli I, Banini M, Greto D, Visani L, Nozzoli F, et al. Re-irradiation for recurrent intracranial meningiomas: Analysis of clinical outcomes and prognostic factors. Radiother Oncol. 2024;195:110271. 10.1016/j.radonc.2024.110271. 38588920 10.1016/j.radonc.2024.110271

[70] Harari CM, Burr AR, Morris BA, Tome WA, Bayliss A, Bhatia A, et al. Pulsed reduced-dose rate re-irradiation for patients with recurrent grade 2 gliomas. Neurooncol Adv. 2024;6(1):vdae073. 10.1093/noajnl/vdae073. 38845694 10.1093/noajnl/vdae073 PMC11154132

[71] Adkison JB, Tome W, Seo S, Richards GM, Robins HI, Rassmussen K, et al. Reirradiation of large-volume recurrent glioma with pulsed reduced-dose-rate radiotherapy. Int J Radiat Oncol Biol Phys. 2011;79(3):835–41. 10.1016/j.ijrobp.2009.11.058. 20472350 10.1016/j.ijrobp.2009.11.058

[72] Witt JS, Musunuru HB, Bayliss RA, Howard SP. Large volume re-irradiation for recurrent meningioma with pulsed reduced dose rate radiotherapy. J Neurooncol. 2019;141(1):103–9. 10.1007/s11060-018-03011-z. 30392090 10.1007/s11060-018-03011-z

[73] Johnson DR, Kimmel DW, Burch PA, Cascino TL, Giannini C, Wu W, et al. Phase II study of subcutaneous octreotide in adults with recurrent or progressive meningioma and meningeal hemangiopericytoma. Neuro Oncol. 2011;13(5):530–5. 10.1093/neuonc/nor044. 21558077 10.1093/neuonc/nor044 PMC3093340

[74] Chamberlain MC, Glantz MJ. Interferon-alpha for recurrent World Health Organization grade 1 intracranial meningiomas. Cancer. 2008;113(8):2146–51. 10.1002/cncr.23803. 18756531 10.1002/cncr.23803

[75] Moazzam AA, Wagle N, Zada G. Recent developments in chemotherapy for meningiomas: a review. Neurosurg Focus. 2013;35(6):E18. 10.3171/2013.10.FOCUS13341. 24289126 10.3171/2013.10.FOCUS13341

[76] Franke AJ, Skelton WI, Woody LE, Bregy A, Shah AH, Vakharia K, et al. Role of bevacizumab for treatment-refractory meningiomas: A systematic analysis and literature review. Surg Neurol Int. 2018;9:133. 10.4103/sni.sni_264_17. 30090665 10.4103/sni.sni_264_17 PMC6057170

[77] Kumthekar P, Grimm SA, Aleman RT, Chamberlain MC, Schiff D, Wen PY, et al. A multi-institutional phase II trial of bevacizumab for recurrent and refractory meningioma. Neurooncol Adv. 2022;4(1):vdac123. 10.1093/noajnl/vdac123. 36225651 10.1093/noajnl/vdac123 PMC9549880

[78] Kaley TJ, Wen P, Schiff D, Ligon K, Haidar S, Karimi S, et al. Phase II trial of sunitinib for recurrent and progressive atypical and anaplastic meningioma. Neuro Oncol. 2015;17(1):116–21. 10.1093/neuonc/nou148. 25100872 10.1093/neuonc/nou148 PMC4483051

[79] Graillon T, Sanson M, Campello C, Idbaih A, Peyre M, Peyriere H, et al. Everolimus and Octreotide for Patients with Recurrent Meningioma: Results from the Phase II CEVOREM Trial. Clin Cancer Res. 2020;26(3):552–7. 10.1158/1078-0432.CCR-19-2109. 31969329 10.1158/1078-0432.CCR-19-2109

[80] Bi WL, Nayak L, Meredith DM, Driver J, Du Z, Hoffman S, et al. Activity of PD-1 blockade with nivolumab among patients with recurrent atypical/anaplastic meningioma: phase II trial results. Neuro Oncol. 2022;24(1):101–13. 10.1093/neuonc/noab118. 34015129 10.1093/neuonc/noab118 PMC8730772

[81] Brastianos PK, Kim AE, Giobbie-Hurder A, Lee EQ, Wang N, Eichler AF, et al. Phase 2 study of pembrolizumab in patients with recurrent and residual high-grade meningiomas. Nat Commun. 2022;13(1):1325. 10.1038/s41467-022-29052-7. 35289329 10.1038/s41467-022-29052-7 PMC8921328

[82] Mirian C, Duun-Henriksen AK, Maier A, Pedersen MM, Jensen LR, Bashir A, et al. Somatostatin Receptor-Targeted Radiopeptide Therapy in Treatment-Refractory Meningioma: Individual Patient Data Meta-analysis. J Nucl Med. 2021;62(4):507–13. 10.2967/jnumed.120.249607. 32859705 10.2967/jnumed.120.249607

[83] Merrell KW, Johnson DR, Steinert KO, Ruff M, Breen W, Gunn HJ, et al. A Prospective, Phase II Study of < sup>177 Lu-Dotatate in Patients with Surgery- and Radiation-Refractory Meningioma: Results of the WHO Grade II/III Cohort. Int J Radiat Oncol Biol Phys. 2024;120(2):S11–2. 10.1016/j.ijrobp.2024.07.003.

[84] Hasenauer N, Muller M, Hanscheid H, Serfling SE, Michalski K, Heinrich M, et al. SSTR-directed peptide receptor radionuclide therapy for recurrent meningiomas: analysis of safety, efficacy and prognostic factors. Eur J Nucl Med Mol Imaging. 2025;53(1):116–27. 10.1007/s00259-025-07336-6. 40455253 10.1007/s00259-025-07336-6 PMC12660467

[85] Kurz SC, Zan E, Cordova C, Troxel AB, Barbaro M, Silverman JS, et al. Evaluation of the SSTR2-targeted Radiopharmaceutical 177Lu-DOTATATE and SSTR2-specific 68Ga-DOTATATE PET as Imaging Biomarker in Patients with Intracranial Meningioma. Clin Cancer Res. 2024;30(4):680–6. 10.1158/1078-0432.CCR-23-2533. 38048045 10.1158/1078-0432.CCR-23-2533

[86] Kurz SC, Rabbee N, Wen PY, Kalapurakal JA, Peereboom DM, Bi LW, et al. TIP-11. MOMENTUM-1: a multicenter, randomized, open-label, phase 2 study of [177Lu]Lu-DOTA-TATE in adults with progressive grade 1–3 meningioma (RTOG 3523). Neurooncology. 2025;27(Supplement5):v422–v. 10.1093/neuonc/noaf201.1671.

[87] 2024. https://clinicaltrials.gov/study/NCT06326190

